# The anatomy of the pelvic plexus in female cadavers: implications for retroperitoneal nerve-sparing surgery

**DOI:** 10.52054/FVVO.16.2.023

**Published:** 2024-06-28

**Authors:** M Mastronardi, D Raimondo, M Mabrouk, A Raffone, M Giorgi, G Centini, E Zupi, R Seracchioli, M Maletta, S Ratti, W.M. O’guin, L Manzoli, A.M. Billi

**Affiliations:** General Surgery Unit, Cattinara University Hospital, 34128 Trieste, Italy; Division of Gynaecology and Human Reproduction Physiopathology, IRCCS Azienda Ospedaliero-Universitaria di Bologna, 40138 Bologna, Italy; Department of Obstetrics and Gynecology, Faculty of Medicine, University of Cambridge, CB2 1TN Cambridge, United Kingdom; Department of Medical and Surgical Sciences, DIMEC, University of Bologna, 40138 Bologna, Italy; Gynecology and Obstetrics Unit, Department of Neuroscience, Reproductive Sciences and Dentistry, School of Medicine, University of Naples Federico II, 80138 Naples, Italy; Department of Molecular and Developmental Medicine, University of Siena, 53100 Siena, Italy; Biomedical and Neuromotor Sciences, Dipartimento di Scienze Biomediche e NeuroMotorie (DIBINEM), University of Bologna, 40138 Bologna, Italy; Department of Cell Biology, New York University School of Medicine, 10016 NY, USA

**Keywords:** Cadavers, pelvic plexus, gynaecologic surgery, nerve-sparing surgery, anatomical landmarks

## Abstract

**Background:**

The inferior hypogastric plexus (IHP) is a crucial structure for female continence and sexual function. A nerve-sparing approach should be pursued to reduce the risk of pelvic plexus damage during retroperitoneal pelvic surgery.

**Objectives:**

To analyse the relationship between the female IHP and several pelvic anatomical landmarks.

**Materials and Methods:**

Standardised cadaveric dissection was performed on 5 nulliparous female cadavers. The relationships of the IHP and the mid-cervical plane (MCP), the mid-sagittal plane (MSP), and the uterosacral ligament (USL) were investigated.

**Main outcome measures:**

Distance between IHP and MCP, MSP, and USL.

**Results:**

Distances between the right IHP and the right MSP (mean distance: 16.3 mm; range: 10.0-22.5 mm) and the right USL (mean distance: 4.8 mm; range: 0-15.0 mm) were shorter than those between the left IHP and ipsilateral landmarks (left MSP distance: 23.5 mm; range 18.0-30.0 mm; left USL distance: 5.0 mm; range: 0-20.0 mm). Although the MCP was 3.3 mm (range: 2.5-4.0 mm) left and lateral to the midsagittal line, the right IHP was closer to the MCP (mean distance: 19.6 mm; range: 13.0-25.0 mm) than the left one (mean distance: 20.2 mm; range: 15.0-26.0 mm).

**Conclusions:**

Distances between the right IHP and the MSP, MCP, and ipsilateral USL, are shorter compared to these associated to the left IHP.

**What is new?:**

Right autonomic pelvic plexus is closer to the midline planes and the ipsilateral USL. These anatomical relationships may be greatly helpful for pelvic surgeon while facing retroperitoneal pelvic surgery and looking for a nerve-sparing approach.

## Introduction

The inferior hypogastric plexus (IHP, or pelvic plexus) plays a key role in female continence and sexual function. Autonomic efferent fibres emerge from its anterior part and innervate the urogenital tract and the inferior part of the rectum, whereas the posterior portion gives rise to fibres directed towards the superior part of the rectum (Centini et al., 2023). The superior branches are responsible for the innervation of the bladder and course along the ureter. The central branches follow the uterine artery to innervate the uterus and the upper vagina. The superior and inferior rectum is innervated by the inferior branches, arising from the posterior and anterior surface of the plexus, respectively. These branches are sympathetic and parasympathetic fibres coursing close to the middle rectal artery (Centini et al., 2023).

By using the “Laparoscopic Neuro-Navigation” technique, Possover et al. (2004) comprehensively analysed the correlation between the localisation and the extension of pelvic nerve fibres damage during laparoscopic surgery and the postoperative morbidity. They divided the IHP into three vertical portions: a cranial portion with a cranio-caudal extension of 3 cm, a mid-portion measuring 2 cm, and a caudal portion, extending 3 caudal to the pouch of Douglas. They showed that damage of the cranial portion of the IHP lead to hypo-anaesthesia of the fornix and the dorsal vaginal cuff; destruction of the mid-portion adds loss of fullness sensation of the bladder and, occasionally, of the rectum; while damage of the caudal portion is responsible for bladder atony alone or bladder and rectum atony combined, depending on the damage located ventrally or laterally to the rectum, respectively (Possover et al., 2004).

Accidental damage of the IHP during retroperitoneal pelvic surgery can be responsible for important visceral dysfunctions, dramatically affecting women’s quality of life (Possover et al., 2004; Kavallaris et al., 2010; Possover and Lemos, 2011; Cosma et al., 2013; Fermaut et al., 2016; Darwish and Roman, 2017; Ripperda et al., 2017). Therefore, it may be useful to identify anatomical landmarks that can help the surgeon to prevent IHP injury and functional sequelae.

In the literature, previous cadaveric studies have evaluated the pathway and the relationship of the IHP with respect to just one anatomical landmark (Ercoli et al., 2003; Fermaut et al., 2016; Ripperda et al., 2017). Furthermore, there is a lack of data concerning the specific position of the IHP in each hemipelvis and the differences between them.

The aim of the present study was to analyse the relationship of the IHP with several anatomical landmarks through cadaveric dissection. We also assessed any anatomical difference concerning position of the IHP between the two hemipelvis.

## Materials and methods

### Study protocol

This was a prospective observational study following an a priori designed study protocol.

Detailed dissections were performed by experts in retroperitoneal anatomy (W.M.O.G., L.M., A.M.B.) on embalmed female cadavers obtained from the ‘Body Program’ at Bologna University (Italy) and New York University (USA), from June 2017 to October 2017. Age, race, height, weight, parity, and cause of death were obtained for all specimens.

The aim of the study was to analyse the anatomical relationships between the IHP and intrapelvic anatomical landmarks including the mid-cervical plane (MCP), the mid-sagittal plane (MSP), and the uterosacral ligament (USL),

All cadavers were dissected using a laparotomic approach. Dissections were carried out on each hemipelvis, without transecting the cadavers in the midline.

The distance between the closest point of the nerves from the IHP to a given anatomical landmark was noted. We considered as anatomical landmarks the MCP, the MSP, and the USL.

**Figure 1:**
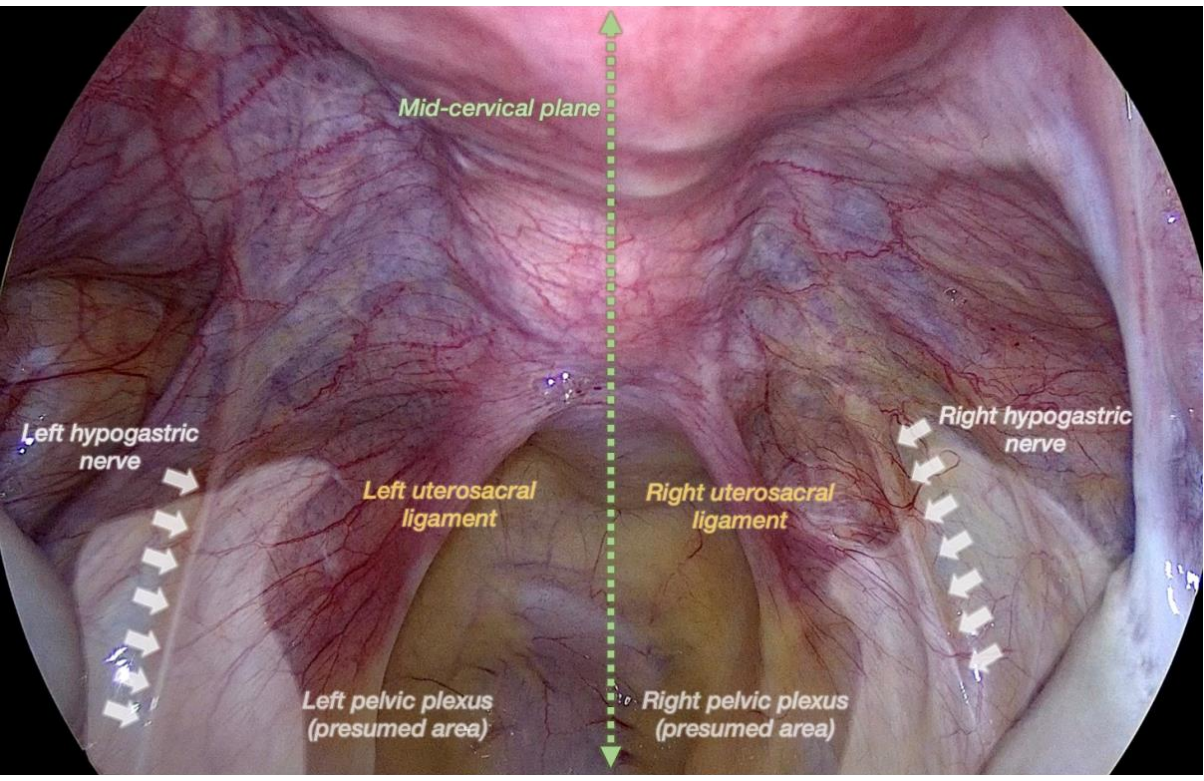
Laparoscopic view of the mid-cervical plane and other pelvic anatomical landmarks.

The MCP is a vertical plane dividing the uterine cervix into right and left halves (Figure 1). In order to identify it, we considered the median vertical line splitting the posterior cervical wall into two parts, passing between the insertion of the uterosacral ligaments on the uterine cervix, and the median vertical line splitting the anterior cervical wall into two parts. The MCP passes through these two lines.

**Figure 2:**
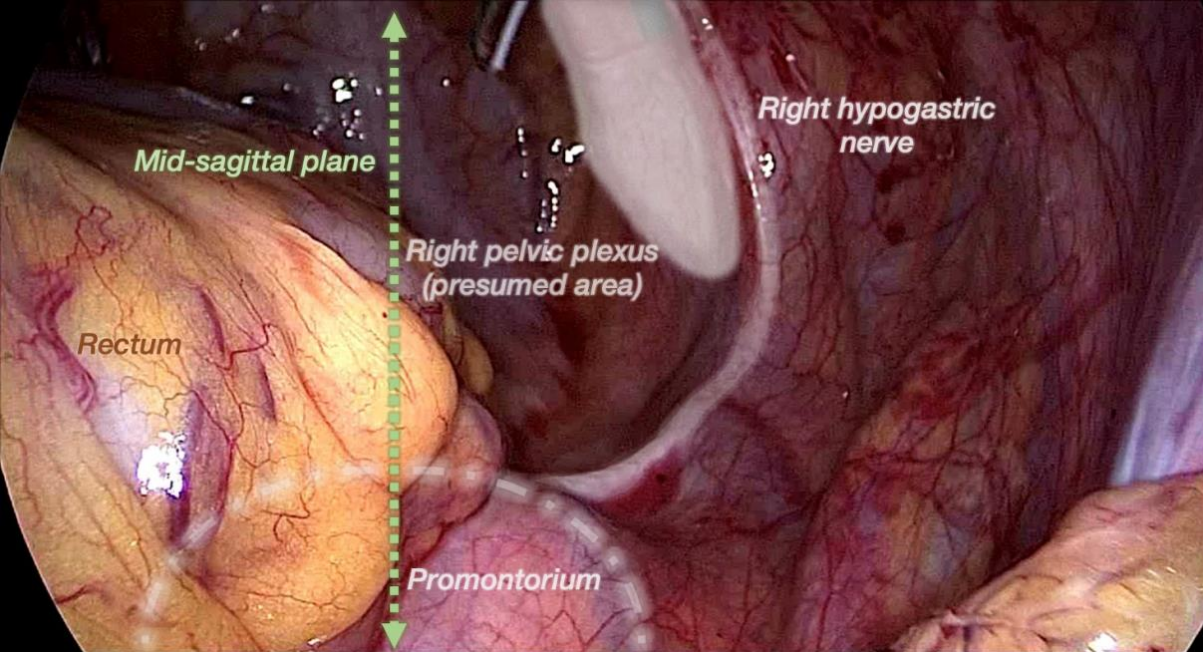
Laparoscopic view of the mid-sagittal plane and other pelvic anatomical landmarks.

The MSP is a vertical plane passing through the midline of the body that divides the latter into right and left halves. In the pelvis, this plane passes through the sacral promontory and the middle point of the pubic symphysis (Figure 2).

Uterosacral ligaments were palpated to identify their midportion at the level of ischial spines.

Each measurement was repeated three times using a ruler. The mean of the three measurements was used for analyses.

Measurements were tabulated; descriptive statistics were used for data analysis and reporting with the use of STATA version 15.1 (StataCorp LP).

### Dissection technique

Before starting the dissection, pelvic anatomical landmarks were identified, such as sacral promontory, ureters, the USL, and their insertion on the uterine cervix.

**Figure 3:**
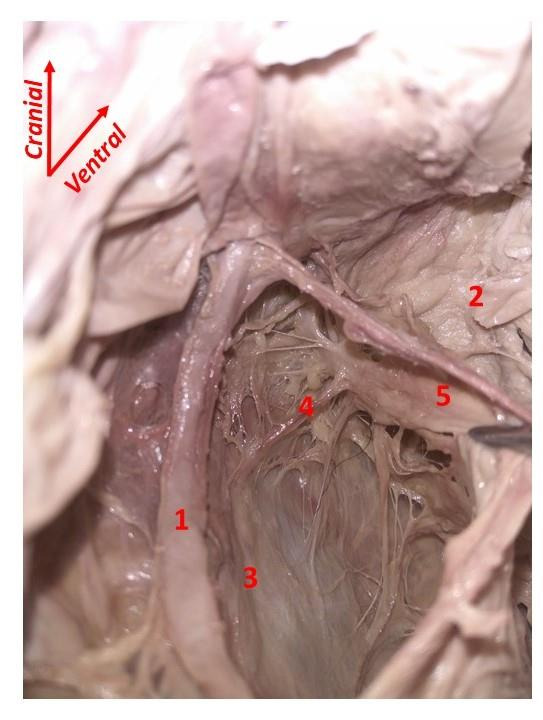
Development of the left medial pararectal space. 1. Ureter; 2. Rectum enveloped by fascia propria recti; 3. Hypogastric nerve; 4. Lateral ligament of the rectum; 5. ‘Hypogastric’ fascia.

The parietal peritoneum was opened at the level of the sacral promontory in order to develop the medial pararectal space, between the ureter and the rectum. We adopted an ‘interfascial technique’ (Moszkowicz and Peschaud, 2014), respecting all the extraperitoneal fascial structures and avoiding nerve injuries as much as possible (Figure 3). In fact, according to Toldts’ law of fascial coalescence (Toldt, 1879), it is possible to identify in the retroperitoneum some avascular and aneural cleavage planes, interposed to completely independent structures, organs, and viscera. To carry out the separation of different fascial layers safely, it is important to understand how these structures are organised around the rectum.

It is possible to summarise their organisation as follow: anterior to the rectum, the rectovaginal fascia separates fascia propria recti from the posterior vaginal wall; one of its lateral continuations separates the pelvic plexus from the mesorectum, connecting dorsally with the ‘hypogastric’ fascia. This fascia is located postero-laterally to the rectum, between the fascia propria recti and the presacral fascia, being ventral to the course of the hypogastric nerve (HN) and the IHP (Federative Committee on Anatomical Terminology, 2001; Kinugasa et al., 2007).

**Figure 4:**
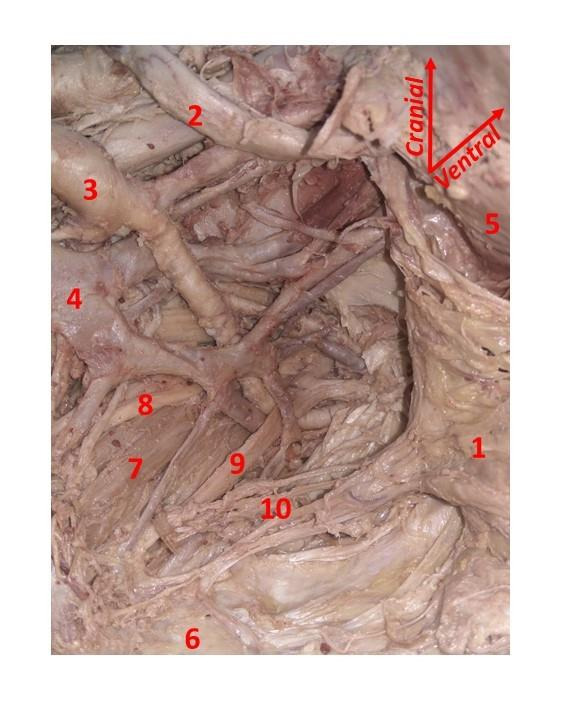
Inferior hypogastric plexus (IHP) on the left hemipelvis. 1. Rectum; 2. Ureter; 3. Internal iliac artery; 4. Internal iliac vein; 5. Uterus; 6. Sacrum; 7. Piriformis muscle; 8. Sacral spinal nerve 1 (S1); 9. Sacral spinal nerve 2 (S2); 10. IHP with branches to the rectum.

**Figure 5:**
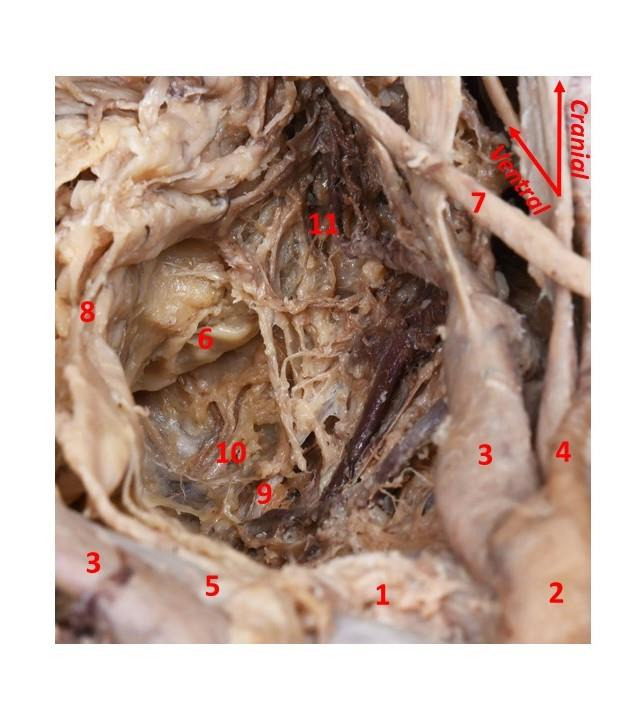
Inferior hypogastric plexus (IHP) on the right hemipelvis. 1. Sacral promontory; 2. Right common iliac artery; 3. Internal iliac arteries; 4. Right external iliac artery; 5. Left common iliac vein; 6. Rectum; 7. Right ureter; 8. Right hypogastric nerves pushed anteriorly to show their contribution to the IHP; 9. Sacral spinal nerve 2 (S2); 10. Sacral spinal nerve 3 (S3); 11. IHP.

After visualisation of the HNs covered by their fascia and the lateral ligament of the rectum, we opened the ‘hypogastric’ fascia to reach the IHP (Figures 4 and 5).

### Ethical statement

The present study was exempted from review by Institutional Review Board, in accordance with the Guidelines of the ‘Body Program’ of Bologna University (Italy) and New York University (USA).

## Results

Cadaveric dissections were performed on a total of five nulliparous embalmed female cadavers. All cadavers were Caucasian with an average age of 55 years (range: 50-60) and body mass index (BMI) of 25.0 kg/m2 (range: 18.0-30.0 kg/ m2). Dissections and available medical histories revealed no obvious signs of pelvic disease.

In all cases the dissection was feasible and allowed identification of the IHP. It appeared as a pyramid-shaped plexus, receiving contributions from HNs, sacral splanchnic nerves, coming from the sacral sympathetic trunk, and pelvic splanchnic nerves, coming from the ventral rami of the second, third and fourth sacral roots (S2, S3 and S4). The sacral sympathetic trunk was found medial to the sacral foramina, getting thinner in its caudal part. However, it was always possible to identify its contribution to the IHP. Almost at the level of the midportion of the USLs, the HNs, and the pelvic splanchnic nerves, coursing toward the postero- lateral side of the rectum, join the pelvic plexus. IHP branches were followed in order to analyse their relationship with the given landmarks.

Anatomical measurements are shown in Table I. The closest distance between branches of the IHP and MCP and MSP were 19.9 mm (range: 13.0-26.0) and 19.9 mm (range: 10.0-30.0), respectively. Branches of the IHP were very close and sometimes corresponded to the USL. The closest mean distance was 4.9 mm (range: 0-20.0).

**Table I:** Anatomical measurements on cadavers.

Anatomical measurement	Mean (range)		
Distance between MCP and MSP	3.3 (2.5-4.0)		
	Right plus Left	Right	Left
Closest distance between IHP and MCP	19.9 (13.0-26.0)	19.6 (13.0-25.0)	20.2 (15.0-26.0)
Closest distance between IHP and MSP	19.9 (10.0-30.0)	16.3 (10.0-22.5)	23.5 (18.0-30.0)
Closest distance between IHP and USL	4.9 (0-20.0)	4.8 (0-15.0)	5.0 (0-20.0)

Data are expressed as mean, in millimetres (range). Abbreviations: IHP, inferior hypogastric plexus; MCP, mid-cervical plane; mm, millimetres; MSP, midsagittal plane; USL, uterosacral ligament.

Concerning differences between the two hemipelvis, the right IHP was observed to be closer to the MSP (mean 16.3 mm; range 10.0-22.5 mm) than the left one (mean 23.5 mm; range 18.0-30.0 mm). Furthermore, the right IHP appeared closer to the ipsilateral USL (mean 4.8 mm; range 0-15.0 mm) than the left one (mean 5.0 mm; range 0-20.0 mm).

Furthermore, although the MCP was found to be 3.3 mm (range 2.5-4.0 mm) to the left of the midsagittal line, the right IHP was closer to the MCP (mean 19.6 mm; range 13.0-25.0 mm) than the left one (mean 20.2 mm; range 15.0-26.0 mm).

## Discussion

Our study focused on the detailed anatomical cartography of the IHP after a careful dissection of the female pelvic retroperitoneum, expanding knowledge on IHP location and the relationships with surrounding structures.

Cadaveric dissection studies are essential for teaching deep pelvic anatomy (Sanguin et al., 2021). The use of a cadaver model along with repeated multistep dissections, and the following transcription of the cadaver dissection enables residents and young surgeons to become familiar with the identification of pelvic nerves, thereby increasing the surgeon’s level of confidence, reducing operating times, and preventing unexpected injuries. Our study tried to extend the traditional topographic anatomy of the IHP anatomy, by analysing the relationship between the IHP and other pelvic landmarks, such as pelvic planes including the MCP, the MSP, and the USL. An accurate knowledge of the differences between the two sides of the pelvis is essential to preserve retroperitoneal structures such as the hypogastric plexus during surgical dissection. Given the wide anatomical variability reflected by our distance ranges, an interfascial approach between fascia propria recti and pre-hypogastric fascia may be the best option to achieve nerve-sparing surgery (Kinugasa et al., 2007; Moawad et al., 2021; Ripperda et al., 2017; Seracchioli et al. 2019; Zakhari et al., 2020).

The IHP is mainly formed by pelvic splanchnic (parasympathetic) and sacral splanchnic (sympathetic) branches; a smaller contribution derives from sympathetic fibres from the lower lumbar ganglia, which descend into the plexus from the superior hypogastric plexus (SHP) through the HNs. Visceral afferent fibres follow the course of sympathetic and parasympathetic fibres to the spinal cord. The IHP gives origin to a network of pelvic branches, which supply the pelvic viscera directly or via periarterial plexuses (Standring, 2015).

In the female, it lies lateral to the uterine cervix, vaginal fornix, and the posterior part of the urinary bladder, often extending into the broad ligaments of the uterus. The upper limit of the plexus corresponds approximately to the level where the uterine artery crosses the ureter in the base of the broad ligament (Azaïs et al., 2013). Branches of the IHP can be divided into three groups: a first group directed to the rectum; a second to the uterus, and a third to the urinary bladder and the vagina (Ercoli et al., 2003). The IHP is responsible for the sensory and motor innervation of the vagina, the vesical bladder, and the rectum (Standring, 2015; Possover et al., 2004; Li et al., 2018; Centini et al., 2023), and its accidental damage during surgery can be responsible for several visceral dysfunctions (Possover et al., 2004; Kavallaris et al., 2010; Cosma et al., 2013; Fermaut et al., 2016; Darwish and Roman, 2017; Ripperda et al., 2017).

Positions of the IHP and its branches in relation with the USL have been described by previous cadaveric studies. On a gross anatomical study, Ripperda et al. (2017) isolated the IHP and its branches. They found the IHP to be lateral and inferior to the midportion of the USL, between the vagina and the rectum, describing that its branches entered the midportion of the USL in 35.5% of the specimens. Since the USL is not a ’real’ ligament but a condensation of connective tissue supporting the uterus (Possover et al., 2005; Ceccaroni et al., 2013), we found a very close relationship between the IHP and the USL. Noteworthy, we observed that on the right side the IHP was closer to the USL and the virtual fusion between them were more frequent on this side. Moreover, we found that on the right side the IHP was also closer to the mid- sagittal and mid-cervical planes. These findings can be probably related to the fact that the SHP and its division into HNs are located at the level of the aortic bifurcation, at the left of the midsagittal line (Centini et al., 2024; Fermaut et al., 2016; Ripperda et al., 2017).

Considering these anatomical relationships, MCP and MSP might become useful landmarks to underscore retroperitoneal pelvic nerves anatomy during gynaecologic surgery (Balaya et al., 2019; Moawad et al., 2021). Indeed, while the IHP is a retroperitoneal structure, the MCP and the MSP are extraperitoneal planes relying on visible (cervix) or palpable (sacrum) structures, hence they are easily recognisable at the beginning of surgery. The IHP position may be inferred from the distances detected in our study.

Nevertheless, our cadaveric study was performed on healthy women, with anatomical relationships likely not being affected by pelvic diseases. The MCP might be displaced by space- occupying diseases such as fibroids, endometriosis, or pelvic organs prolapse, while the MSP could be difficult to assess in overweight patients. However, reaffirming the didactic value of the identified anatomical relationships, both the MCP and the MSP could also serve as valuable landmarks in clinical practice, allowing for optimal pelvic dissection and anatomical restoration in challenging surgeries.

Our data showed that the USL seems to be a good landmark because the nerves are very close to it, especially on the right side. According to Ripperda et al. (2017), these findings can explain the occurrence of postoperatively de novo bladder, bowel, or sexual dysfunction after USL transection during surgery. USL anatomy may be completely distorted by endometriosis of the posterior compartment or by apical prolapse (DeLancey, 2016; Koninckx et al., 2021), which may affect the distances discovered through our cadaveric study and limit the clinical reproducibility of our data. The USL may be extremely elongated by pelvic organ prolapse from the IHP or can become fused to the IHP by endometriotic nodules (Aurore et al., 2020; DeLancey, 2016). Indeed, our study aims to enhance understanding of pelvic anatomy and nerves, highlighting the intricate correlations between anatomical landmarks, particularly for young surgeons (Centini et al., 2023; Moawad et al., 2021; Zakhari et al., 2020). In our opinion, traditional anatomical landmarks employed during nerve-sparing surgery, such as the deep uterine vein (Robin et al., 2022; Robin et al., 2024), could be complemented by utilising correlations between pelvic structures and planes, as shown in our study.

A notable strength of this study is its prospective design. In order to avoid artifacts, the distances of the IHP from the selected anatomical landmarks were obtained through the development of the plane between fascia propria recti and the ‘hypogastric’ fascia, anteriorly to the presacral fascia, followed by the opening of the ‘hypogastric’ fascia, in order to identify the IHP behind it (Seracchioli et al., 2019).

However, our data must be further confirmed due to the small number of cases and exclusion of some conditions like previous pregnancy, pelvic organ prolapse, and history of pelvic surgery that can alter basic fascial configuration.

## Conclusions

The right autonomic pelvic plexus is closer to midline planes and the ipsilateral USL. These anatomical relationships may be greatly helpful for pelvic surgeon while facing retroperitoneal pelvic surgery and looking for a nerve-sparing approach. The MCP, the MSP, and the USL may be easily identifiable anatomical landmarks during surgery. Clinical studies are needed to confirm the reproducibility and applicability of our data, especially during laparoscopic surgery.
